# Radiotherapy with or without chemotherapy for patients with T1-T2 glottic carcinoma: retrospective analysis

**DOI:** 10.1186/1758-3284-2-20

**Published:** 2010-07-30

**Authors:** Naoki Hirasawa, Yoshiyuki Itoh, Shunichi Ishihara, Seiji Kubota, Junji Itoh, Yasushi Fujimoto, Tsutomu Nakashima, Shinji Naganawa

**Affiliations:** 1 Department of Radiology, Nagoya University Graduate School of Medicine, 65 Tsurumai-cho, Shouwa-ku, Nagoya 4668550, Aichi, Japan; 2 Department of Otolaryngology, Nagoya University Graduate School of Medicine, 65 Tsurumai-cho, Shouwa-ku, Nagoya 4668550, Aichi, Japan

## Abstract

**Background:**

To assess the results for local control (LC) and survival in patients with early-stage glottic cancer (GC) who were treated by radiotherapy (RT) with or without chemotherapy.

**Methods:**

Fifty-eight patients with T1-T2 squamous cell carcinoma of the glottis who were treated between 2001 and 2006 were analyzed retrospectively. Potential prognostic factors for LC were evaluated by univariate analysis.

**Results:**

The 5-year LC rate in all patients was 84.3%. The overall 5-year LC rates for patients with T1a, Tb, and T2 GC were 85.9%, 83%, and 85%, respectively. Of the 58 patients, eight developed recurrent disease at the primary site, and one had lymph node recurrences on the neck. In the final analysis, the total laryngectomy-free survival rate was 93% at five years, and the ultimate LC rates for T1a, Tb, and T2 were 100%, 90.9%, and 95.2%, respectively. In a univariate analysis of 55 patients, there was no statistical significance between the LC rate for RT alone and that for chemoradiation. Only two patients died of laryngeal carcinoma, and one died of intercurrent disease. Fifty-five patients were living disease-free at the end of the study period. The 5-year overall survival (OS) rate for all patients was 88.1%, and the 5-year OS rates for T1a, Tb, and T2 were 91.6%, 77.8%, and 89.9%, respectively.

**Conclusions:**

The retrospective analysis showed a high rate of LC and larynx preservation in patients with T1-T2 GC by means of RT with or without chemotherapy. There was, however, no statistical difference in LC rates for the two types of therapy.

## Background

Glottic carcinoma (GC), the most common laryngeal cancer, and is usually detected early because of the symptomatic occurrence of hoarse voice. The recommended strategies for early GC with the intent of larynx preservation are radiation therapy (RT), transoral laser therapy, and partial laryngectomy [1,2].

RT is the preferred therapeutic method in early GC. The advantages of RT in terms of preservation of the structure and function of the larynx have been documented extensively, and RT currently is the initial treatment of choice in most institutions, with surgery being reserved as a salvage option for local failure. For T1 GC, the LC rate for RT alone has been reported to be about 80-90% [3-8], whereas for T2 GC, the LC rate has been about 65-80% [4,5,7,8]. Based on our previously reported data for 1990-1997, the LC rate of T2 GC by RT alone was 65% at our institute.

The purpose of this study was to review retrospectively our experience in the treatment of T1 and T2 GC through RT with or without chemotherapy from 2001 to 2006.

## Methods

### Patient characteristics

A retrospective review was performed of 58 patients who had undergone radical RT with or without chemotherapy to the larynx for Stage I-II GC (T1-T2, N0 according to the 2002 International Union Against Cancer classification system). Patients were treated at Nagoya University Hospital between January 2001 and April 2006. All patients received RT as the first-choice treatment. Patient inclusion criteria were a histologic diagnosis of infiltrative squamous cell carcinoma and no previous RT for head and neck neoplasms. Fifty-five patients (95%) were male, and three (5%) were female. The median age was 64 years (range, 44-92 years). The initial examinations before the start of the treatment included physical examination, blood count, biochemical examination, electrocardiography, and staging procedures including computed tomography (CT) scan of the whole body and upper endoscopy. The presence of human papillomavirus (HPV) was not examined. Table 1 contains a summary of the clinical characteristics of the 58 patients who were included in the study. Twenty-four patients had tumors classified as T1a, 13 had T1b tumors, and 21 patients T2 tumors. All patients were followed for a median period of 48 months (range, 13-84 months) or until death.

**Table 1:** Patients characteristics

	n	percentage
Total no. of patients	58	100
Age median (range)	64 y.o. (44-92)	
Male/Female	55/3	95/5
Performance status (ECOG)		
0-1	56	96
2	2	4
Histology		
squamous cell carcinoma	58	100
Stage		
T1a	24	41
T1b	13	22
T2	21	36

### Treatments detail

#### Radiotherapy

Table 2 contains a summary of the treatment courses of the 58 patients. Thirty-nine patients were treated with RT alone; 19 received RT and chemotherapy. Fifty-seven patients were treated with parallel-opposed fields using 4-MV, and only one patient was treated using 6-MV. Fifty-four patients received a continuous course of RT with a once-daily fraction of 2 Gy, and four patients were treated with a continuous course of RT delivered twice a day to a total dose of 74 Gy to 82 Gy, with 1.2 Gy to 1.3 Gy per fraction. Wedge filters of 15 or 30 degrees were used to optimize the dose distribution to achieve a homogeneity of ± 5%. The field size ranged from 25 to 36 cm^2^. No prophylactic neck irradiation was performed in any of the cases. All patients were immobilized using a thermoplastic mask during treatment.

**Table 2:** Radiation therapy

	n	percentage	
Conventional	54	93	
Dose range	18-82Gy	Median	70Gy/35fx

AFX-CB	3	5	
Dose range	60.8-76Gy	Median	67Gy

Accelerated hyperfractionation	1	2	
Dose range	60Gy	Median	60Gy/40fx

AFX-CB: accelerated fractionation with concomitant boost

#### Chemotherapy

The various chemotherapy drugs and regimens used are listed in Table 3. The chemotherapeutic regimen for five patients given a high dose of CDDP/5-FU consisted of continuous infusion of 5-FU at a dose of 700 mg/m^2^/day on days 1-4, combined with a 2-hr infusion of CDDP at a dose of 70 mg/m^2^/day on day 1. Low-dose CDDP/5-FU was continuously administered to five other patients via different routes through a catheter placed in the central vein. The daily dose of 5-FU was given at 200 mg/m^2^, and that of CDDP was 4 mg/m^2^. CDDP and 5-FU were administered for 24 hr every day, except Saturday and Sunday, from the day irradiation was started. The regimen for four patients was a low dose of CDDP alone, consisting of 60-min administration of CDDP at a dose of 5 mg/body after RT. The other regimens are listed in Table 3.

**Table 3:** Chemotherapy and these regimen

	With chemotherapy	Radiation alone	total
	n (%)	n (%)	n
T1a	1 (4)	23 (96)	24
T1b	4 (30)	9 (70)	13
T2	14 (67)	7 (33)	21

**Regimen**	n (%)		

Low dose CDDP	4 (21)		
Low dose CDDP/5FU	5 (26)		
High dose CDDP/5FU	5 (26)		
Carboplatin	2 (10)		
Alternative CDDP/5FU	2 (10)		
UFT(oral antidrug)	1 (5)		

In three patients treated with chemoradiotherapy (two cases) or RT alone (one case), tumor responses were very poor. For these patients, treatments were discontinued at 18 Gy, 36 Gy, and 52 Gy, and partial laryngectomies were performed.

In our basic concept, the regimens of a low dose of CDDP, Carboplatin, UFT, or a low dose of CDDP/5-FU were mainly used for bulky T1 and T2 tumor, on the other hand, the regimens of a high dose of CDDP or alternative CDDP/5-FU were used for unfavorable T2 tumor (maybe, near T3).

### Statistical Analysis

LC and total laryngectomy-free survival were assessed from the beginning of RT until evidence of recurrence or until laryngectomy. Survival curves were calculated using the method of Kaplan and Meier, and log-rank tests were used to define the statistical significance of the observed differences in survival and LC based on univariate analysis. The results were considered statistically significant at the level of *P *< 0.05. In the univariate analysis, the variables analyzed included age (63 > vs. ≤ 63), T category (T1 vs. T2), overall treatment time (> 49 vs. ≤ 49), and chemotherapy (combined. vs. not combined).

### Follow-up

After RT alone or combined with chemotherapy, the patients were evaluated at 1-month intervals for the first year, at 2-month intervals during the second year, every 3 months during the third year, every 4 months during the fourth year, and every 6 months thereafter. The patients who presented with recurrence of disease in the follow-up time submitted to salvage treatment by total or partial laryngectomy. The interval time between these events was recorded.

## Results

### Local control and patterns of failure

For all 58 patients, the 5-year LC rate was 84.3%. The 5-year LC rates for T1a, Tb, and T2 were 85.9%, 83%, and 85%, respectively. The difference between the sub-stage LC rates was not statistically significant.

Of the 58 patients, eight developed recurrent disease at the primary site, and one had lymph node recurrences on the neck. In three of the eight patients, tumor responses were very poor. The therapies of these three patients were discontinued at 18 Gy (bulky T1b), 36 Gy (T2), and 52 Gy (T2), and the patients underwent partial laryngectomy. Four of five patients with local recurrence submitted to salvage surgery; one refused surgery. Thus, only one case was uncontrolled. In the final analysis, the total laryngectomy-free survival rate was 93% at 5 years, and the ultimate LC rates for T1a, Tb, and T2 were 100%, 90.9%, and 95.2%, respectively (Table 4).

**Table 4:** Local control rate and survival according to the T stage

	T1a	T1b	T2
5-year local control rates without surgery	85.9%	83%	85.1%
5-year local control rates after surgery	100%	90.9%	95.2%
5-year Overall survival	91.6%	77.8%	89.9%
5-year Cause-specific survival	100%	87.5%	95.2%

### Overall survival (OS) and cause-specific survival (CSS) rates

The 5-year OS rate for all patients was 88.1%, and the 5-year OS rates for T1a, Tb, and T2 were 91.6%, 77.8%, and 89.9% respectively. The 5-year CSS rate in the 58 patients was 95.8%, and the 5-year CSS rates for T1a, Tb, and T2 were 100%, 87.5%, and 95.2%, respectively (Table 4).

### Univariate analysis

The variables analyzed in 55 patients included age, T category, overall treatment time, and chemotherapy for LC (Table 5). Results of univariate analysis showed no statistical significance for any of the variables.

**Table 5:** Univariate Analysis (in 55 patients)

parameter	5-year local control rate(%)	P-value
Stage		
T1N0 (n = 36)	86.8	0.51
T2N0 (n = 19)	94.1	
Overall treatment times		
> 49 days (n = 27)	91.4	0.88
≤ 49 days (n = 28)	87.7	
Chemotherapy		
combined (n = 17)	93.7	0.52
none (n = 38)	86.5	
Age		
> 63 y.o. (n = 27)	92.5	0.75
≤ 63 y.o. (n = 28)	86.1	

### Complications

There were no severe acute complications. No late complications such as chondronecrosis were seen, and no patients required hospitalization due to complications.

### Second primary cancers

Twelve of the 58 patients (20%) had second primary cancers, and two patients (3%) had third cancers. Moreover, 75% of the second lesions were in the upper aerodigestive tract. Second and third primary sites are shown in Table 6. There were five cancers diagnosed synchronously with glottic cancer. The 5-year survival rate in 12 patients with double primary cancers was 74.0%, and that in 46 patients without double or triple cancers was 92.5% (p = 0.18).

**Table 6:** Incidence of secondary primary cancers in this study

Primary site	number	percentage
thyroid	2	15
oropharynx	1	7.7
hypopharynx	1	7.7
esophagus	2	15
stomach	2	15
lung	3	20
liver	1	7.7
colon	3	20

Total	15	100

## Discussion

The goals of treatment for early GC include cure and laryngeal voice preservation.

In this study, the 5-year LC rate for all patients was 84.3%, and the 5-year LC rates for T1a, Tb, and T2 were 85.9%, 83%, and 85%, respectively. The difference between the sub-stage LC rates was not statistically significant. These results are favorable. In particular, the outcome of LC for T2 GC was better than our previous results (1990-1997) as mentioned in introduction, however, in the univariate analysis for combined chemotherapy for LC, the difference had no statistical significance (p = 0.52). The 5-year LC rate with chemoradiation was 93.7% (Table 5).

According to recently published guidelines [1,2] for head and neck cancer treatment, all patients with T1-T2 laryngeal cancer should be treated, at least initially, with the intent of larynx preservation. Chemoradiotherapy is not recommended for early GC. However, several analyses of the risk of local failure after RT for early GC have shown the probability of success to be closely related to the volume or bulk of the lesion [9-11]. Moreover, recent studies have shown an improvement in LC for patients with T1 and T2 GC when total radiation is delivered in a shorter overall treatment time with a high-dose fractionation [4,12] or hyperfractionation schedule [13-15]. Therefore, we treated several cases on a hyperfractionation schedule during the early period of this analysis.

In contrast, several other reports [16-20] have indicated that chemoradiation for T2GC is promising and that LC rates are higher than those for RT alone in Japan.

Therefore, we conducted chemoradiation for bulky T1 and T2 GC with the intent to improve LC. The 5-year LC rate with chemoradiation was 93.7%. The result was favorable, but the difference had no statistical significance.

Our study had several limitations. First, we were unable to determine the best combination of therapies using anticancer drugs because this study was not prospective and various chemotherapy regimens were used at different times. Second, therapies of three patients were discontinued due to poor responses, and these cases were counted as recurrences in the statistical analyses. ****Two patients, one with a bulky T1b tumor and the other with an unfavorable T2 tumor, responded poorly in spite of chemoradiation with low-dose CDDP and 5-FU. Perhaps bulky tumors and unfavorable T2 tumors require more intensive and powerful anticancer drugs. Because many regimens of chemotherapy were used in the cases presented in this study, consideration must be made of the choice of anticancer drugs, and a prospective trial must be conducted in which chemoradiation is used on an optimal dosing schedule for T1 bulky or T2 GC. A phase II study of chemoradiotherapy with S-1(per oral anti-cancer drug) for T2GC is currently underway.

Many recent studies have suggested novel markers of radiosensitivity, such as DNA ploidy [21], expression of epidermal growth factor [22], p53 [22,23], Bcl-2 [24], and microvessel density [25]. Regarding microvessel density, the biological effects of ionizing radiation are critically dependent on the existence of oxygen in tissues, which may be another reason for the poor responses. The cases of poor response are considered to involve radioresistant tumors, said to account for 6-30% of all tumors [25]. If poor responses to radiotherapy with or without chemotherapy can be predicted, surgery or another procedure may be selected.

In the present study, a second malignancy occurred in more than 20% of the cases, 75% of which were in the upper aerodigestive tract. Additionally, the prognoses for patients with a second malignancy were poorer than those of patients with a single malignancy, though the difference was not statistically significant.

After treatment is completed, regular check-ups are very important, and long-term follow-up is required [26,27].

## Conclusions

By means of radiotherapy with or without chemotherapy, we achieved a high rate of LC in patients with T1-T2 GC. Although the combination therapy yielded the most favorable results, there was no statistical difference in the LC rates.

## Competing interests

The authors declare that they have no competing interests.

## Authors' contributions

NH and YI designed the study, acquired and interpreted the data, and wrote the manuscript. SI, SK, JI, YF, TN and SN contributed to study design, interpretation of data. SN revised the manuscript. All authors have given final approval of the version to be published.
